# Symptom burden in community-dwelling older people: temporal trends in the Helsinki Aging Study

**DOI:** 10.1007/s40520-021-01918-8

**Published:** 2021-07-03

**Authors:** T. E. Lehti, H. Öhman, M. Knuutila, H. Kautiainen, H. Karppinen, R. Tilvis, T. Strandberg, K. H. Pitkälä

**Affiliations:** 1 Department of General Practice and Primary Health Care, University of Helsinki, Helsinki, Finland; 2 Social Services and Health Care, Helsinki, Finland; 3 Geriatric Medicine, University of Helsinki and Helsinki University Hospital, Helsinki, Finland; 4 Center for Life Course Health Research, University of Oulu, Oulu, Finland; 5 Primary Health Care Unit, Helsinki University Hospital, Helsinki, Finland; 6 Tukholmankatu 8 B, Biomedicum 2 B, 00290 Helsinki, Finland

**Keywords:** Symptom burden, Multimorbidity, Temporal trends, Aged, Psychological wellbeing, Oldest-old

## Abstract

**Background:**

Changes in older people’s symptoms across recent decades have not been investigated.

**Aims:**

We analyzed temporal trends in symptom burden by comparing data from independent, cross-sectional cohorts retrieved in 1989, 1999, 2009, and 2019. Furthermore, we compared the association between symptom burden and psychological wellbeing (PWB) in older men and women.

**Methods:**

The Helsinki Aging Study recruited a random sample of people aged 75, 80, and 85 in 1989, and random samples aged 75, 80, 85, 90, and 95 in 1999, 2009, and 2019 (four study waves). Altogether, 6263 community-dwelling people answered the questions concerning symptoms in the questionnaire surveys. The symptoms inquired in all study waves were dizziness, back pain, joint pain, chest pain, shortness of breath, and loss of appetite. Symptom burden was calculated according to the number of symptoms and their frequency (score range: 0–6). PWB and the Charlson comorbidity index were calculated.

**Results:**

Symptom burden decreased in both men and women aged 75 and 80 from 1989 to 2019. Changes in cohorts aged 85 + were nonsignificant. There was a significant difference in symptom burden between men and women in all ages with men having fewer symptoms. PWB decreased with increasing symptom burden. Men had greater PWB than women up to severe levels of symptom burden.

**Conclusions:**

Symptom burden decreased from 1989 to 2019 in cohorts aged 75–80, whereas changes remained nonsignificant in cohorts aged 85 +. To our knowledge, this is the first study to examine temporal trends in symptom burden.

## Introduction

The numbers of people surviving to old age are increasing worldwide. Conflicting hypotheses were made in the late twentieth century on whether health and quality of life will improve or deteriorate for the growing older population. Gruenberg proposed in 1977 that, thanks to better control of infectious diseases, more individuals with chronic diseases would survive to older age, a theory he entitled “the failures of success”[1], while Fries predicted a postponed onset of chronic illness and a “compression of morbidity”[2]. Recent cohort studies looking at temporal trends in health and functioning in the older population mostly support Fries’ hypothesis. They have shown a decrease in disabilities [3, 4] as well as improvements in cognitive functioning [4], psychological wellbeing (PWB) [5], and self-rated health [6] in later born cohorts. Contrasting evidence has also emerged that shows increased disease prevalence and loss of mobility function in the older population in recent years [7]. To our knowledge, symptoms and their overall burden have not been examined in the older population in this respect.

Symptom burden denotes the multidimensional burden of symptoms that an individual is experiencing. It has recently attracted research attention as an interesting self-reported measure of health in the aging population. Symptom burden has been shown to be associated with comorbidities [8–10], functional status [8–10], self-rated health [10], prognosis [9–11] and quality of life [10, 12]. Symptom burden is most often calculated by summing up points from a list of symptoms. Previous studies have used various symptom burden grading instruments such as the PRIME-MD [11], the Edmonton Symptom Assessment Scale [8], the SSS-8 Somatic symptom scale [13], and the Memorial Symptom assessment scale [14, 15], as well as others [9, 10, 16]. Our prior study as well as others have used instruments that only include somatic symptoms in the symptom burden score [10, 11, 13], while others have included psychiatric symptoms such as anxiety and depressed mood [8, 9, 14–16]. Even though these instruments vary both in which symptoms they contain and whether they grade the severity or frequency of symptoms, the results of these studies are in line with each other, providing evidence that symptom burden is an independent determinant of relevant health-related outcomes such as prognosis and self-rated health. Pain and tiredness/lack of energy have been among the most prevalent symptoms in several previous cohort studies [8, 9, 14, 16].

We have recently examined the validity of a symptom scale which included eight symptoms chosen by experienced geriatricians. This symptom burden scale was associated with psychological wellbeing and it predicted mortality [10]. In addition, it showed sufficient internal consistency. Symptoms have been inquired in the Helsinki Aging Study for 30 years, and they have been found relevant and easy to understand for older people.

Even if evidence is accumulating about the importance of symptom burden in wellbeing and prognosis, we lack information on its development over time in the older population. The aim of this study was to analyze temporal trends in symptom burden in people aged 75–95 using Helsinki Aging Study data of four cross-sectional cohorts that span 30 years from 1989 to 2019. The Helsinki Aging Study enables comparisons between people of the same age groups born and surveyed 10–30 years apart. We also compared the association between symptom burden and PWB in older men and women. To our knowledge, this is the first study to look at temporal trends in symptom burden.

## Methods

### Study design and participants

The Helsinki Aging Study (1989–present) is a series of population-based, cross-sectional cohort studies designed to examine comorbidities, health, and functioning in the community-dwelling older population in Finland [17, 18]. Every 10 years since 1989, a questionnaire survey has targeted independent, cross-sectional (transverse) cohorts of people aged 75 +. This study examines Helsinki Aging Study questionnaire data from 1989, 1999, 2009, and 2019.

The study cohorts were retrieved from the Finnish Population Information System at four time points in 1989, 1999, 2009, and 2019. In 1989, the study recruited random samples of 300 community-dwelling persons from age groups 75 and 80 as well as 298 persons aged 85 (total *n* = 898). In 1999, 1000 persons in each age group of 75, 80, and 85 were recruited as well as all 90-year-olds and 95-year-olds (*n* = 734 and *n* = 187, respectively; total *n* = 3,921). In 2009, 600 persons in each age group of 75, 80, 85 and 90 were recruited as well as all 95-year-olds (*n* = 233; total *n* = 2,633). In 2019, 600 persons in each age group of 75, 80, 85 and 90 were recruited as well as all 95-year-olds (*n* = 389; total *n* = 2,789). A reminder was sent to those who did not respond the first time.

Due to delays in posting the questionnaire and in the Finnish Population Information System, some of the recruited people had died, moved away or moved to institutionalized care before receiving the questionnaire. This was taken into account when estimating the response rates. The estimated response rates were 93% in 1989 to 80% in 1999, 73% in 2009, and 74% in 2019.

The Helsinki University Hospital Ethics Committee approved the study design.

### Measures

#### Participant characteristics

Age and sex were extracted from the participants’ Finnish national personal identification numbers. Marital status was self-reported in the questionnaire (“Are you married or cohabiting/unmarried/divorced or separated/widowed?”). Common medical diagnoses were listed, and participants provided a yes/no answer to each: diabetes, hypertension, coronary heart disease, myocardial infarction, cardiac failure, hypercholesterolemia, stroke, dementia, ventricular or duodenal ulcer, chronic bowel disease, chronic obstructive pulmonary disease, asthma, rheumatoid arthritis, osteoarthritis, other musculoskeletal disease, psychiatric disorder (e.g. depression), prostatic hyperplasia, cancer (if yes, specify which), sleep apnea, and some other chronic disease (if yes, specify which). We calculated the Charlson comorbidity index [19] using these self-reported medical diagnoses.

#### Outcomes

Participants were invited to report in the questionnaire whether they had experienced any of a list of symptoms over the past 2 weeks on a three-step scale: never, sometimes, or daily. In our analyses, we examined a set of six symptoms that were included in all four questionnaires from 1989 to 2019: (1) dizziness, (2) joint pain that hinders activity, (3) back pain that hinders activity, (4) loss of appetite, (5) chest pain or discomfort in the chest, and (6) shortness of breath. The participants who did not respond to any of the six symptom items were excluded from further analyses. The rest, who had given an answer (never, sometimes, or daily) to at least one symptom item, were included. The six-item symptom scale was closely similar to the eight-item scale validated by our previous study [10] with the exception of two symptoms, leg pain when walking and urinary incontinence, which were not present in the questionnaire survey in all four study waves.

Similar to our previous study [10], symptom burden was defined as a weighted sum of all reported symptoms. If a symptom was experienced sometimes, it added 0.5 point, while experiencing a symptom daily added 1 point to the sum. Thus, symptom burden could have values between zero and six.

PWB was calculated using the previously validated PWB score that looks at six dimensions of PWB [20, 21]: (1) satisfaction with life, (2) positive life orientation, (3) feeling of being needed, (4) plans for the future, (5) loneliness, and (6) feelings of depression. Respondents were asked to give yes/no answers to the first four questions and a graded response (seldom or never/sometimes/often or always) to the last two. Answers pointing to a more positive life orientation yielded a higher score; yes/no would yield 1/0 point, and graded questions would yield 0, 0.5, or 1 point according to the answer. The points were summed and then divided by the number of questions the respondent answered to get the PWB score (range 0–1).

### Statistical analysis

The data are presented as means with standard deviation (SD) or as counts (*n*) with percentages (%). Statistical significances for the hypothesis of linearity across categories of cohorts (study year) were evaluated using the Cochran-Armitage test for trend, analysis of variance or logistic models with an appropriate contrast. Symptom burden was adjusted for age, sex, and the Charlson comorbidity index when testing the hypothesis of linearity. PWB was reported as means with 95% confidence intervals for each subgroup. The PWB means were adjusted for age and the Charlson comorbidity index. In the case of violation of the assumptions (e.g., non-normality), a bootstrap-type test was used. The normality of the variables was tested using the Shapiro–Wilk W test. The Stata 16.1 (StataCorp LP; College Station, Texas, USA) statistical package was used for the analysis.

## Results

Table 1 presents cohort characteristics from 1989 to 2019. The first cohort in 1989 was the smallest with 556 participants after excluding those who had not answered any symptom items. The proportion of men increased from 27% in 1989 to 56% in 2019. The first cohort in 1989 had the largest proportion of participants under the age of 80 (39%), while the proportion of participants aged ≥ 90 was significantly larger in 2009 and 2019 than in 1999—approximately 30% in 2009 and 2019 (Table 1). We have combined the 90- and 95-year-old cohorts in the analyses.

**Table 1:** Characteristics of cross-sectional cohorts in 1989, 1999, 2009, and 2019

	1989 *n* = 556	1999 *n* = 2473	2009 *n* = 1583	2019 *n* = 1651	*p* value*
Women, *n* (%)	405 (73)	1762 (71)	1089 (69)	1064 (64)	< 0.001
Age, *n* (%)					< 0.001
75	219 (39)	701 (28)	386 (24)	405 (25)	
80	186 (33)	674 (27)	378 (24)	399 (24)	
85	151 (27)	617 (25)	349 (22)	367 (22)	
90–95	0 (0)	481 (19)	470 (30)	480 (29)	
Widowed, *n* (%)	247 (45)	1125 (47)	658 (42)	586 (36)	< 0.001
Depressed, *n* (%)					< 0.001
Never	353 (66)	1419 (64)	983 (66)	1121 (70)	
Sometimes	164 (31)	685 (31)	468 (31)	450 (28)	
Often or always	19 (4)	117 (5)	41 (3)	40 (2)	
Charlson comorbidity index^a^, mean (SD)^b^	1.4 (1.3)	2.1 (2.0)	2.0 (1.8)	1.7 (1.6)	< 0.001
Dementia, *n* (%)	471 (20)	188 (13)	218 (13)	471 (20)	< 0.001
Coronary heart disease, *n* (%)	118 (23)	606 (26)	344 (24)	314 (19)	< 0.001
COPD/asthma, *n* (%)	94 (18)	428 (18)	232 (16)	192 (12)	< 0.001
Somatic symptoms, *n* (%)					
Dizziness	199 (36)	817 (33)	507 (32)	464 (28)	< 0.001
Joint pain	254 (46)	1071 (43)	749 (47)	799 (48)	0.005
Back pain	242 (44)	979 (40)	660 (42)	698 (42)	0.40
Loss of appetite	96 (17)	394 (16)	243 (15)	251 (15)	0.26
Chest pain	159 (29)	676 (27)	360 (23)	273 (17)	< 0.001
Dyspnea	245 (44)	1066 (43)	427 (27)	369 (22)	< 0.001
Symptom burden, mean (SD)	1.39 (1.12)	1.31 (1.18)	1.23 (1.17)	1.11 (1.08)	< 0.001**

*p for linearity

**p for linearity adjusted for age, sex, and the Charlson comorbidity index [19]

^a^Charlson et al. 1987[19]

^b^SD = standard deviation of the mean

The number of widowed participants decreased over time: 45% of participants in 1989 compared to 36% in 2019. The proportion of participants who reported being depressed sometimes or often/always decreased (Table 1). The Charlson comorbidity index initially climbed from 1.4 on average in 1989 to 2.1 in 1999, followed by a decrease to 1.7 in 2019 (*p* for linearity < 0.001). The prevalence of coronary heart disease and COPD/asthma was lowest in the latest study wave in 2019, while the prevalence of dementia was higher in 2019 than in two previous study waves (Table 1).

Symptom burden decreased linearly from a mean 1.39 in 1989 to 1.11 in 2019 (*p* for linearity < 0.001 adjusted for age, sex, and the Charlson comorbidity index). Joint pain and back pain were among the most common symptoms in all cohorts from 1989 to 2019 with 40–44% of participants experiencing back pain and 43–48% experiencing joint pain. Dyspnea was highly prevalent (over 40%) in the cohorts in 1989 and 1999 but decreased to 27% in 2009 and 22% in 2019 (*p* < 0.001). The other two symptoms that showed a statistically significant decline in prevalence from 1989 to 2019 were dizziness and chest pain: from 29 to 17% for chest pain and from 36 to 28% for dizziness (*p* for linearity < 0.001 for both).

**Fig. 1:**
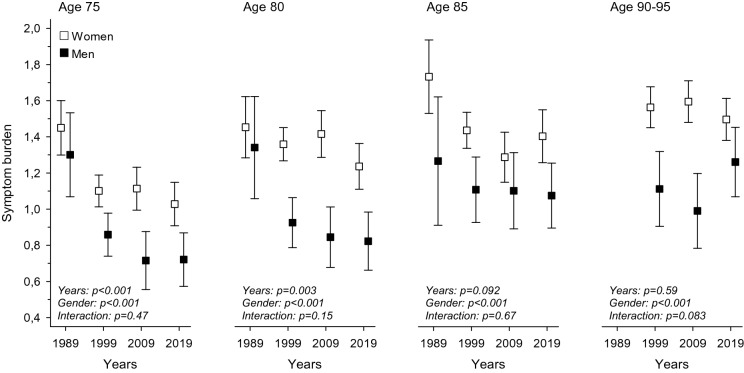
Average symptom burden from 1989 to 2019 in cross-sectional age cohorts (75, 80, 85, and 90–95) for women and men separately: symptom burden means with 95% confidence intervals adjusted for the Charlson comorbidity index [19]

Figure 1 shows the development of mean symptom burden from 1989 to 2019 in each age cohort for women and men separately. There is a significant difference in symptom burden between men and women in all age groups. Symptom burden decreased for both men and women aged 75 and 80 from 1989 to 2019. No clear time trend was seen for the 85- and 90 + -year-olds.

**Fig. 2:**
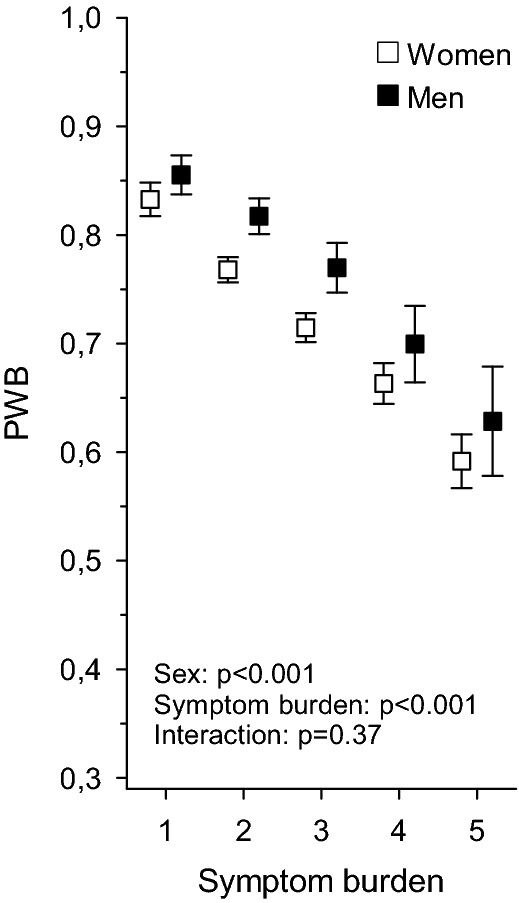
The relationship between symptom burden (0–6) and PWB in men and women in all cohorts combined: PWB means with 95% confidence intervals adjusted for age and the Charlson comorbidity index [19]

Figure 2 depicts the relationship between symptom burden and PWB in men and women, respectively. A negative linear relationship is seen for both sexes with men having, on average, higher levels of PWB.

## Discussion

This study shows a significant decrease in somatic symptom burden in 75- and 80-year-old cohorts from 1989 to 2019. No similar change is seen for the oldest-old cohorts (85 +). Of individual symptoms, dyspnea, chest pain, and dizziness showed a statistically significant decrease across decades. It needs to be noted that the proportion of oldest-old participants and the proportion of men were larger in 2009 and 2019 than the previous rounds. Men generally had fewer symptoms and greater wellbeing than women. However, even after adjusting for age, sex and the Charlson comorbidity index, there was an overall decreasing trend in symptom burden. PWB decreased linearly with increasing symptom burden in both sexes.

Like time trends in health and functioning [3–6], symptom burden shows a decreasing trend in our study cohorts over the last 3 decades. Similarly, the Charlson comorbidity index has decreased in the cohorts from 1999 to 2019. However, the change in the number of diagnosed diseases and various symptoms does not automatically imply a reduction or increase in morbidity. The detection of diseases varies in time not only according to diagnostic methods and their availability but also with the introduction of preventive and therapeutic novelties (e.g., in cardiovascular diseases, osteoporosis, pain, and dementia) or lack of them.

We have recently shown that symptom burden is linearly associated with PWB [10]. In this study, we confirm this finding for both women and men separately and show that men enjoy higher levels of PWB up to severe levels of symptom burden. Our finding is in line with previous research showing that women report more symptoms and use healthcare services more frequently than men [22]. More research is needed to explain this difference.

Symptom burden is associated with prognosis and wellbeing [10] and, therefore, plays a significant role in older people’s quality of life and health. It is comforting to see that symptom burden is decreasing in the later born cohorts at the same time as comorbidities and depressive symptoms have decreased. Our results suggest prolonged wellbeing in the aging population.

This study has the following strengths. First, we have collected representative cohort data at four time points spanning three decades, making this the first study to analyze time trends in symptom burden. Symptom burden is important since it has independent prognostic significance irrespective of comorbidities [10]. Second, we have targeted an equivalent population in each research round. Third, the same survey instrument was used for all cohorts. The response rate remained good in all rounds, although it reduced from 93% in 1989 to 74% in 2019.

The study also has weaknesses. We have looked at cross-sectional data of independent, separate samples, and, therefore, cannot draw conclusions on causality. The symptom scale does not take into account the severity of symptoms, only their frequency. In addition, the inquired symptoms cannot be directly connected to diseases. Instead, we wanted to explore older people’s subjective experiences which have been shown to be relevant as regards prognosis in prior studies [8–11]. The age and sex distributions in each research round were different with a higher percentage of oldest-old participants and men in the most recent rounds. However, the overall decreasing trend in symptom burden remained significant even when adjusting for age, sex, and comorbidities. We have only looked at home-dwelling older people, which constitutes a possible bias. Significantly fewer older people enter institutionalized care now than in the late twentieth century, as more assistive services are increasingly brought to homes. This can explain the higher prevalence of dementia among community-dwelling older people in 2019 than in 1999 and 2009. Finally, various confounding factors can be observed at different points of time: societal changes may affect people’s attitudes toward their health and physical symptoms, advances in assistive devices and living conditions may affect how people perceive their health and symptoms, and diagnostic intensity may be the main determinant of the number of diagnoses a person gets.

## Conclusion

We conclude that a decreasing trend in symptom burden can be seen for 75- and 80-year-old cohorts for both women and men from 1989 to 2019. This is the first study to report time trends in symptom burden among community-dwelling older people, and more research is needed to confirm this finding in other populations. Furthermore, we show that older men have, on average, better PWB than older women up to high levels of symptom burden. More research is needed to explore ways to support psychological wellbeing, especially for aging women.

## Data Availability

The datasets generated during and analyzed during the current study are available from the corresponding author on reasonable request.
